# Comparison of Quadratic- and Median-Based Roughness Penalties for Penalized-Likelihood Sinogram Restoration in Computed Tomography

**DOI:** 10.1155/IJBI/2006/41380

**Published:** 2006-04-19

**Authors:** Patrick J. La Rivière, Junguo Bian, Phillip A. Vargas

**Affiliations:** Department of Radiology, The University of Chicago, Chicago, IL 60637, USA

## Abstract

We have compared the performance of two different penalty choices for a penalized-likelihood sinogram-restoration strategy we have been developing. One is a quadratic penalty we have employed previously and the other is a new median-based penalty. We compared the approaches to a noniterative adaptive filter that loosely but not explicitly models data statistics. We found that the two approaches produced similar resolution-variance tradeoffs to each other and that they outperformed the adaptive filter in the low-dose regime, which suggests that the particular choice of penalty in our approach may be less important than the fact that we are explicitly modeling data statistics at all. Since the quadratic penalty allows for derivation of an algorithm that is guaranteed to monotonically increase the penalized-likelihood objective function, we find it to be preferable to the median-based penalty.


Hindawi Publishing Corporation
International Journal of Biomedical Imaging
Volume 2006, Article ID 41380, Pages 1–7
DOI 10.1155/IJBI/2006/41380

### Comparison of Quadratic- and Median-Based Roughness Penalties for Penalized-Likelihood Sinogram Restoration in Computed Tomography

**Patrick J. La Rivière, Junguo Bian, and Phillip A. Vargas**

*Department of Radiology, The University of Chicago, Chicago, IL 60637, USA*

Received 13 December 2005; Revised 3 March 2006; Accepted 5 March 2006

We have compared the performance of two different penalty choices for a penalized-likelihood sinogram-restoration strategy we have been developing. One is a quadratic penalty we have employed previously and the other is a new median-based penalty. We compared the approaches to a noniterative adaptive filter that loosely but not explicitly models data statistics. We found that the two approaches produced similar resolution-variance tradeoffs to each other and that they outperformed the adaptive filter in the low-dose regime, which suggests that the particular choice of penalty in our approach may be less important than the fact that we are explicitly modeling data statistics at all. Since the quadratic penalty allows for derivation of an algorithm that is guaranteed to monotonically increase the penalized-likelihood objective function, we find it to be preferable to the median-based penalty.

Copyright © 2006 Patrick J. La Rivière et al. This is an open access article distributed under the Creative Commons Attribution License, which permits unrestricted use, distribution, and reproduction in any medium, provided the original work is properly cited.

### 1. INTRODUCTION

We have recently developed penalized-likelihood approaches to the problems of sinogram smoothing and sinogram restoration in computed tomography [1–4], with a particular eye to the low-dose regime being considered for screening studies for lung and colon cancer [5–9]. In both cases, we assume that the statistics of each detector measurement are given by the sum of a compound Poisson term, representing the photon counting statistics for polychromatic photons, and a Gaussian term, representing electronic noise. In the case of sinogram restoration, we generalize the measurement model to include blurring coefficients representing sinogram degradations such as off-focal radiation, detector afterglow, and detector crosstalk [2, 4]. From the noisy, degraded measurements, we then seek to estimate a set of "ideal," undegraded line integrals by iteratively maximizing an objective function comprising a sum of a simple Poisson likelihood (an approximation to the measurement statistics assumed above) and a roughness penalty. The estimated line integrals can then be fed into an existing analytic reconstruction algorithm, such as those typically implemented in hardware on commercial CT scanners. The hope is that this iterative sinogram-domain approach would provide some of the statistical advantages of fully iterative approaches to image reconstruction at a lower computational cost.

In our previous studies of the smoothing and restoration approaches, we have made use of a quadratic roughness penalty applied in the line integral (log) domain to the difference between a given sample in sinogram space and its four adjoining neighbors, that is, between a given detector channel and its two neighboring channels (we assumed a single-row detector), as well as to its own reading at the preceding and following view angles. While the approaches performed better in resolution-variance studies at low doses than did Hsieh's noniterative adaptive trimmed mean (ATM) filter [10], the ATM filter was surprisingly effective at reducing the influence of a small number of very noisy measurements without unduly compromising resolution. The ATM filter is only applied to measurements whose signal strength falls below a certain threshold, and it entails replacing the value in question with the trimmed mean of the values in a neighborhood of the sinogram around the measurement in question. The trimmed mean filter is a median-like filter based on order statistics, and it varies adaptively between applying a true median filter and a simple boxcar filter, depending on the signal level. The relatively strong performance of this filter suggested that it would be worthwhile to explore the use of a median-based roughness penalty in the context of the sinogram smoothing and restoration methods we have developed.

The use of median-based roughness penalties in fully iterative penalized-likelihood image reconstruction was pioneered by Alenius et al., who referred to them as median root priors (MRPs) [11, 12]. Unfortunately, it does not appear to be possible to derive an iterative algorithm that is guaranteed to monotonically increase an objective function based on the MRP of Alenius et al. However, they derive a heuristic update equation, based on Green's one-step-late (OSL) strategy [13], that does not necessarily correspond to the maximization of a predefined objective function, but that does yield good results in practice.

In this work, we explore the use of a standard MRP penalty like those of Alenius et al. in the context of our sinogram-restoration approach and we make use of the heuristic, OSL strategy to derive the iterative update. We compare the images qualitatively and quantitatively to those obtained by use of our method with quadratic roughness penalties, as well as to those obtained by use of Hsieh's ATM filter.

### 2. METHODS

### 2.1. Measurement model

We assume that the CT scan produces a set of measurements that are represented as a one-dimensional (1D) vector $\mathbf{y}^{\text{meas}}$, with elements $y_i^{\text{meas}}$, $i = 1, \ldots, N_y$, where $N_y$ is the total number of measurements in the scan, and the index $i$ denotes a particular attenuation line through the patient (i.e., a specific combination of detector channel, detector row, and projection angle).

To review the model, we have been employing [2–4], we assume, when we simulate data, that each $y_i^{\text{meas}}$ is a realization of a random variable $Y_i^{\text{meas}}$ whose statistics are described by

$$Y_i^{\text{meas}} = G_i \sum_{m=1}^{M} E_m \text{Poisson}\left\{ \sum_{j=1}^{N_y} b_{ij} I_j \lambda_m^{(j)} \exp\left[ -\int_{L_j} \mu(\mathbf{x}, E_m)\, dl \right] + s_m^{(i)} \right\} + \text{Normal}\,\{d_i, \sigma_i^2\}, \tag{1}$$

with the various terms in this equation defined in Table 1. The compound Poisson distribution in the first term has been derived and validated by Whiting [14] and rederived by Elbakri and Fessler [15, 16]. We assume that $I_i$, $G_i$, $d_i$, $\sigma_i^2$, the average energy $\overline{E}_i \equiv \sum_{m=1}^{M} E_m\, \lambda_m^{(i)}$ of the incident beam, and an energy-averaged and normalized estimated scatter term $\bar{s}_i \equiv (1/\overline{E}_i) \sum_{m=1}^{M} E_m\, s_m^{(i)}$ are all known.

Our goal is to estimate a set of ideal, "monochromatic" attenuation line integrals:

$$l_i^{(\text{mono})} \equiv \int_{L_i} \mu(\mathbf{x}, E_r)\, dl, \tag{2}$$

$i = 1, \ldots, N_y$, at some reference energy $E_r$ (usually $\overline{E}_i$), from the set of measurements $y_i^{\text{meas}}$, $i = 1, \ldots, N_y$. These estimated line integrals can then be input to a standard analytic reconstruction algorithm as mentioned above.

Our strategy for estimating the line integrals entails maximizing a penalized-likelihood objective function. Because the model of (1) does not yield a tractable likelihood, we approximate it by defining a vector $\mathbf{y}$ of new adjusted measurements with elements

$$y_i \equiv \left[ \left( \frac{y_i^{\text{meas}} - d_i}{\overline{E}_i G_i} \right) + \frac{\sigma_i^2}{G_i^2 \overline{E}_i^2} \right]_+, \tag{3}$$

where $[x]_+$ is $x$ for positive $x$ and zero otherwise, that are realizations of random variables $Y_i$ which we assume are approximately Poisson-distributed:

$$Y_i \sim \text{Poisson}\left\{ \sum_{j=1}^{N_y} I_j b_{ij} e^{-l_j^{(\text{poly})}} + \bar{s}_i + \frac{\sigma_i^2}{G_i^2 \overline{E}_i^2} \right\}, \tag{4}$$

where

$$l_j^{(\text{poly})} = f_j\left( l_j^{(\text{mono})} \right), \tag{5}$$

with $f_j(l)$ being an empirically determined function, typically polynomial, that adequately captures the effect of beam hardening in slices that do not contain substantial amounts of bone [17]. Our strategy is then to estimate the vector $\mathbf{l}^{(\text{poly})}$, with elements $l_j^{(\text{poly})}$, from the vector of adjusted measurements $\mathbf{y}$, since the needed $l_i^{(\text{mono})}$ can then be obtained by inverting (5). For simplicity, we drop the (poly) superscripts from $\mathbf{l}^{(\text{poly})}$ in what follows.

### 2.2. Quadratic penalty approach

Our general strategy thus far [2–4] has been to maximize a penalized-likelihood objective function

$$\Phi(\mathbf{l}; y) \equiv \mathcal{L}(\mathbf{l}; y) - \beta R(\mathbf{l}), \tag{6}$$

where

$$\mathcal{L}(\mathbf{l}; \mathbf{y}) = \sum_{i=1}^{N_Y} y_i \log\left[ \sum_{j=1}^{N_y} I_j b_{ij} e^{-l_j} + r_i \right] - \left[ \sum_{j=1}^{N_y} I_j b_{ij} e^{-l_j} + r_i \right] \tag{7}$$

is the Poisson log-likelihood for the random variables of (4) and where we have defined $r_i \equiv s_i + \sigma_i^2/(G_i^2 \overline{E}_i^2)$.

The roughness penalty $R(\mathbf{l})$ can be expressed in a general form as

$$R(\mathbf{l}) = \sum_{k=1}^{K} \psi_k \left( \sum_{j=1}^{N_y} t_{kj} l_j \right), \tag{8}$$

given by Fessler [18], where $\psi_k$ is a potential function that assigns a cost to the $K$ combinations of attenuation line integral values represented by the linear combinations $\sum_{j=1}^{N_y} t_{kj} l_j$.

Our quadratic penalty approach entails choosing $\psi_k(t) = \omega_k t^2/2$ and constructing the $t_{kj}$ to create differences of a sinogram sample with its horizontal and vertical neighbors, with

Table 1: Definition of terms in (1).

| Variable | Meaning |
|---|---|
| $G_i$ | Detector gain |
| $E_m$ | Energy of $m$th spectral bin |
| $b_{ij}$ | Degradation coefficients |
| $I_i$ | Number of incident photons along $i$th attenuation line |
| $\lambda_m^{(i)}$ | Probability of a photon incident on $i$th attenuation line belonging to $m$th spectral bin |
| $L_i$ | Designates $i$th attenuation line |
| $s_m^{(i)}$ | Number of scattered photons of energy $E_m$ contributing to measurement $i$ |
| $\mu(\mathbf{x}, E)$ | Energy-dependent attenuation map, with $\mathbf{x}$ being spatial coordinate in patient |
| $d_i$ | Dark current in $i$th measurement |
| $\sigma_i^2$ | Electronic noise in $i$th measurement |

$\omega_k = 1/2$ for those neighbors. This is equivalent to

$$R(\mathbf{l}) = \frac{1}{4}\sum_{j=1}^{N_y}\sum_{k\in\mathcal{N}_j}(l_j - l_k)^2, \tag{9}$$

where $\mathcal{N}_j$ denotes the neighborhood comprising the 4 nearest horizontal and vertical neighbors of measurement $j$.

We have derived an algorithm that generates a sequence of estimates of $\mathbf{l}$ that are guaranteed to increase the objective function of (6) by making use of the optimization transfer principal [18], in which at each iteration one defines a surrogate to the likelihood function, such that the vector of line integrals maximizing this surrogate is guaranteed to have a higher penalized likelihood than the previous vector estimate. The resulting update is

$$l_j^{(n+1)} = \left[l_j^{(n)} - \frac{n_j + \beta\sum_{k=1}^{K}\sum_{i=1}^{N_Y} t_{kj}t_{ki}\omega_k l_i^{(n)}}{c_j^{(n)} + \beta v_j}\right]_+, \tag{10}$$

where the notation $l_j^{(n)}$ denotes the estimate of $l_j$ after the $n$th iteration. Here,

$$n_j = \sum_{i=1}^{N_Y} I_j b_{ij}\dot{g}_i\left(\sum_{j'=1}^{N_y} I_{j'}b_{ij'}e^{-l_{j'}^{(n)}} + r_i\right)e^{-l_j^{(n)}}, \tag{11}$$

where

$$\dot{g}_i(x) = y_i/x - 1, \qquad v_j \equiv \sum_{k=1}^{K}|t_{ki}|t_k\omega_k, \quad \text{with } t_k \equiv \sum_{i=1}^{N_y}|t_{ki}|, \tag{12}$$

and the $c_j^{(n)}$ are the curvatures of paraboloidal surrogates constructed to give rise to an overall, easy-to-maximize quadratic surrogate to the objective function. One choice for the curvatures that guarantee monotonicity is

$$c_j^{(n)} = I_j\sum_{i=1}^{N_y} b_{ij}, \tag{13}$$

although in practice we make use of a different set of curvatures that do not guarantee monotonicity but that in practice lead to faster convergence [19].

### 2.3. Median penalty

For the median penalty approach, we employ a more heuristic approach based on producing a sequence of estimates that, in the absence of a penalty, would yield a maximum-likelihood estimate, but that incorporates a penalty term in each iteration that discourages deviations from the local median of the last iteration. This is consistent with the approach of Alenius et al. in fully iterative reconstruction [11, 12]. Specifically, the update is defined as

$$l_j^{(n+1)} = \arg\max_{l_j>0}\left\{S(\mathbf{l},\mathbf{l}^{(n)}) - \beta R^{(\text{med})}(\mathbf{l},\hat{\mathbf{m}}^{(n)})\right\}, \tag{14}$$

where $S(\mathbf{l},\mathbf{l}^{(n)})$ is a surrogate to the log likelihood to be described below and

$$R^{(\text{med})}(\mathbf{l},\hat{\mathbf{m}}^{(n)}) \equiv \frac{1}{2}\sum_{k=1}^{N_y}\frac{\left(l_k - \hat{m}_k^{(n)}\right)^2}{\hat{m}_k^{(n)}} \tag{15}$$

is the median penalty, with

$$\hat{m}_k^{(n)} = \text{Median}\,(\mathbf{l}^{(n)}, \mathcal{N}_k) \tag{16}$$

denoting the median of the values of $\mathbf{l}^{(n)}$ in some neighborhood $\mathcal{N}_k$ around value $k$.

The surrogate $S(\mathbf{l},\mathbf{l}^{(n)})$ is defined as

$$S(\mathbf{l},\mathbf{l}^{(n)}) \equiv \sum_{k=1}^{N_y} S_k\left(l_k,\mathbf{l}^{(n)}\right), \tag{17}$$

where

$$S_k(l_k,\mathbf{l}^{(n)}) \equiv \sum_{i=1}^{N_y}\frac{I_k b_{ik}e^{-l_k^{(n)}}}{\overline{y}_i^{(n)}}g_i\left(\frac{e^{-l_k}}{e^{-l_k^{(n)}}}\overline{y}_i^{(n)}\right) + \frac{r_i}{\overline{y}_i^{(n)}}g_i\left(\overline{y}_i^{(n)}\right), \tag{18}$$

with

$$\overline{y}_i^{(n)} \equiv \sum_{k=1}^{N_y} I_k b_{ik}e^{-l_k^{(n)}} + r_i, \tag{19}$$

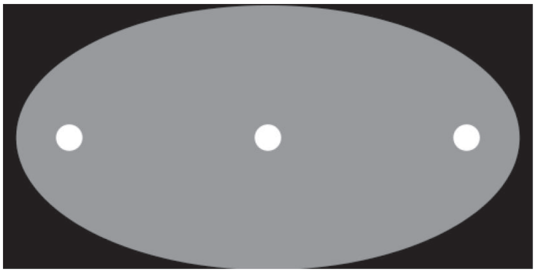

Figure 1: Illustration of the numerical ellipse phantom used for resolution-noise studies.

and $g_i(x) \equiv y_i \log x - x$. This surrogate satisfies both $S(\mathbf{l}, \mathbf{l}^{(n)}) \leq L(\mathbf{l})$, $\forall l_j \geq 0$ and $S(\mathbf{l}^{(n)}, \mathbf{l}^{(n)}) = L(\mathbf{l}^{(n)})$, and thus in the absence of the penalty term, finding the $\mathbf{l}$ maximizing this surrogate necessarily increases the likelihood [4].

Substituting (17) and (15) into (14), our update is given by

$$l_j^{(n+1)} = \arg\max_{l_j>0} \left\{ \sum_{k=1}^{N_y} S_k\left(l_k, \mathbf{l}^{(n)}\right) - \beta \frac{1}{2} \sum_{k=1}^{N_y} \frac{\left(l_k - \widehat{m}_k^{(n)}\right)^2}{\widehat{m}_k^{(n)}} \right\}. \quad (20)$$

We can solve for the maximum by setting the derivative with respect to $l_j$ equal to zero. Doing so yields

$$-\sum_{i=1}^{N_Y} I_j b_{ij} \dot{g}_i \left( \frac{e^{-l_j}}{e^{-l_j^{(n)}}} \overline{y}_i^{(n)} \right) e^{-l_j} - \beta \left( \frac{l_j^{(n)} - \widehat{m}_j^{(n)}}{\widehat{m}_j^{(n)}} \right) = 0. \quad (21)$$

Solving for $l_j$ yields the update

$$l_j^{(n+1)} = \left[ -\ln \left( \frac{\sum_{i=1}^{N_Y} I_j b_{ij} \left( \left( y_i / \overline{y}_i^{(n)} \right) e^{-l_j^{(n)}} \right) + \beta \left( \left( l_j^{(n)} - \widehat{m}_j^{(n)} \right) / \widehat{m}_j^{(n)} \right)}{\sum_{i=1}^{N_Y} I_j b_{ij}} \right) \right]_+ . \quad (22)$$

### 3. RESULTS

### 3.1. Qualitative results

To compare the two penalties to each other as well as to the ATM filter, we simulated projections of a numerical ellipse phantom shown in Figure 1 that we have used previously and that is modeled on the physical phantom employed by Hsieh [10]. We computed a sinogram of 1024 angles $\times$ 1024 bins of extent 0.5 mm at the isocenter with a source-to-isocenter distance of 540.0 mm. We simulated the data according to the forward model of (1) with discretization of a realistic CT spectrum into 1 keV bins. We assumed that the phantom was water-equivalent except for the three circular structures, which we assumed to be bone.

We simulated two exposure levels: a clinically typical $I_i = 2.5 \times 10^6$ and a low-dose level $I_i = 2.5 \times 10^5$. We chose $G_i = 3.57 \times 10^{-3}$ pA/keV, such that $G_i \overline{E}_i = 0.25$ pA/quanta for $\overline{E}_i = 70$ keV, and $\sigma_i^2 = 10.0\,\text{pA}^2$ for all $i$. We included the effects of off-focal radiation in the simulation by convolving with kernels having 13 nonzero values arrayed diagonally with slope $-2$ in discrete sinogram space. The central value (corresponding to the zero point of the kernel) had relative value 1.0 and the six values on either side had relative value 0.02 (these thirteen values were normalized so that their sum was 1.0). We found through simulations with water-equivalent phantoms that the beam-hardening effect of the simulated tube spectrum was well represented as a second-order polynomial $f(l) = l - 0.007l^2$.

We reconstructed images by means of four different approaches.

(1) Hanning. Simple discrete deconvolution of the effect of off-focal radiation, followed by beam-hardening correction and fan-beam filtered backprojection (FFBP) reconstruction with a Hanning filter of varying cutoffs.
(2) ATM. Presmoothing of data by means of the ATM filter, followed by simple discrete deconvolution of the effect of off-focal radiation, beam-hardening correction, and reconstruction by FFBP with an unapodized ramp filter. The ATM filter was implemented with cutoff parameter $\lambda = 75.0$ pA and baseline parameter $\delta = 0.05$ pA, as in [10] by Hsieh, and with the filter length varying from 3 to 19.
(3) Quadratic. Penalized-likelihood sinogram restoration using the quadratic neighborhood penalty followed by beam-hardening correction and reconstruction by FFBP with an unapodized ramp filter. The smoothing parameter $\beta$ was varied from 0.01 to 50.
(4) Median. Penalized-likelihood sinogram restoration using the quadratic neighborhood penalty followed by beam-hardening correction and reconstruction by FFBP with an unapodized ramp filter. The smoothing parameter $\beta$ was varied from 0.01 to 50. The size of the neighborhood used in median calculation was $3 \times 3$.

Figure 2 shows typical results of these reconstructions, where we have selected values of the smoothing parameters for the ATM and penalized-likelihood images that give approximately matched resolution at the center insert and approximately matched noise levels at the right insert, based on the resolution-variance results to be described below. It can be

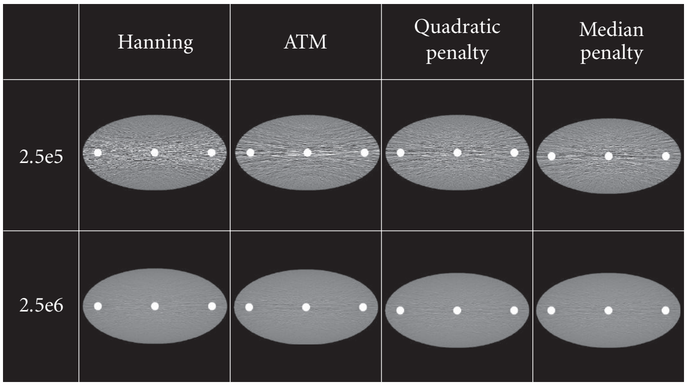

FIGURE 2: The left column illustrates reconstructions by FFBP employing a Hanning filter with cutoff 0.8 times the Nyquist frequency. The second column illustrates reconstructions by FFBP after sinogram smoothing by the ATM filter method and off-focal radiation deconvolution. The third column illustrates reconstructions by FFBP after sinogram restoration by the quadratic penalty method. The final column illustrates reconstructions by FFBP after sinogram restoration by the median penalty method. Exposures are listed at left. The window width is 400 and the level is 40.

seen that the noise level in the low-dose data leads to severe streaking artifacts in the Hanning filter reconstruction and that these are suppressed by the approaches under consideration, more so for the penalized-likelihood approaches than for the ATM approach. The results all appear to perform similarly at the higher-dose level. These qualitative impressions were explored quantitatively by use of resolution-variance studies.

### 3.2. *Resolution-variance tradeoffs*

To characterize resolution, we determined the local edge-spread function at the central and right high-attenuation inserts. The vertical profiles through these structures have profiles that are well fit by error functions parametrized by width $\sigma_b$ which implies that the effective blurring kernel is Gaussian with standard deviation $\sigma_b$. We employ the FWHM of the Gaussian, 2.35 $\sigma_b$, as our measure of resolution. To obtain an accurate fit, we performed targeted reconstructions of the central and right high-attenuation inserts with 0.25 mm pixel size from 10 different noise realizations. We then averaged the reconstructions together to obtain relatively low-noise profiles on which to perform the fitting. We characterized noise by calculating the average standard deviation of the pixel values in circular regions of interest (ROIs) of diameter 16.0 mm placed adjacent to, but not overlapping, the central and right high-attenuation inserts. We then plotted the resulting noise measure versus the resolution measure for the same location. Images reconstructed after smoothing with different values of the smoothing parameters $\alpha$ or filter length $\beta$ provide different combinations of such values and allowed us to sweep out a resolution-noise curve.

The results are given in Figure 3, where it can be seen that at the low-dose level, the two penalized-likelihood-based approaches both outperform the ATM filter in terms of resolution-noise performance. They perform very similarly to each other, with perhaps a slight advantage to the quadratic penalty. At high-dose levels, the approaches all perform relatively similarly.

### 4. CONCLUSIONS

We have compared two different penalty choices for a penalized-likelihood sinogram-restoration strategy we have been developing, one is a quadratic penalty we have employed previously and the other is a median-based penalty. We compared the approaches to a noniterative adaptive filter that loosely but not explicitly models data statistics. We found that the two approaches produced very similar resolution-variance tradeoffs to each other and that they outperformed the ATM filter in the low-dose regime, which suggests that the particular choice of penalty in our approach may be less important than the fact that we are explicitly modeling data statistics at all.

It is not possible to conclude, of course, that the penalized-likelihood approaches would outperform any noniterative adaptive filter. In generating the resolution-variance tradeoffs for the ATM filter, we made use of the parameters $\lambda$ and $\delta$ given by Hsieh in describing the filter in [10] and varied the filter length parameter $\beta$. The filter length $\beta$ is the most natural parameter to vary in sweeping out resolution-variance curves, but it is possible that further adjusting the parameters $\lambda$ and $\delta$ could further improve the achievable tradeoffs. In particular, it is possible that the version of the ATM filter implemented on GE scanners has been optimized beyond what was presented in [10].

Since the quadratic penalty allows for derivation of a monotonic algorithm guaranteed to increase the likelihood function while the median filter approach offers no such guarantee, we find it to be preferable to the median-based penalty. However, it might be worthwhile to explore the new class of median-like prior proposed by Hsiao et al. that does indeed involve maximization or minimization of a joint objective function involving the image of interest and an

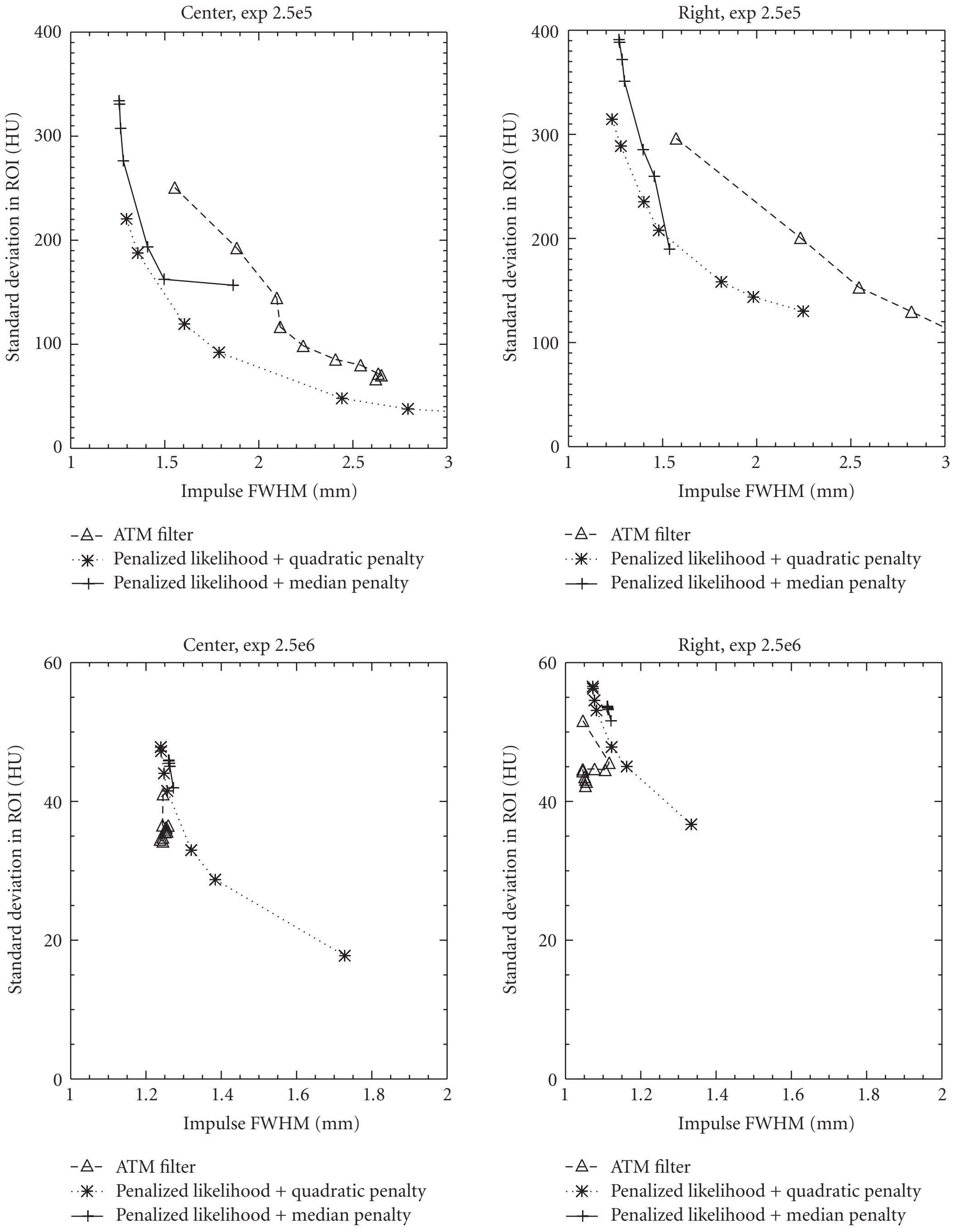

Figure 3: Resolution-noise tradeoffs for exposures $2.5 \times 10^5$ and $2.5 \times 10^6$ at the center and right circular inserts in the ellipse phantom for the three approaches under consideration.

auxiliary field derived from the local medians of the image [20]. Another possibility for future work would be to explore a penalty that uses Hsieh's ATM filter as an OSL prior much as the median prior was used here.

### ACKNOWLEDGMENT

This work was supported by the Schweppe Foundation.

### REFERENCES

[1] P. J. La Rivière and D. M. Billmire, "Reduction of noise-induced streak artifacts in X-ray computed tomography through spline-based penalized-likelihood sinogram smoothing," *IEEE Transactions on Medical Imaging*, vol. 24, no. 1, pp. 105–111, 2005.

[2] P. J. La Rivière, "Penalized-likelihood sinogram restoration for CT artifact correction," in *Proceedings of the IEEE Nuclear*

*Science Symposium and Medical Imaging Conference (NSS-MIC '04)*, vol. 5, pp. 3303–3307, Rome, Italy, October 2004, CD-ROM.

[3] P. J. La Rivière, "Penalized-likelihood sinogram smoothing for low-dose CT," *Medical Physics*, vol. 32, no. 6, pp. 1676–1683, 2005.

[4] P. J. La Rivière, J. Bian, and P. Vargas, "Penalized-likelihood sinogram restoration for computed tomography," to appear in *IEEE Transactions on Medical Imaging*.

[5] S. Sone, S. Takashima, F. Li, et al., "Mass screening for lung cancer with mobile spiral computed tomography scanner," *Lancet*, vol. 351, no. 9111, pp. 1242–1245, 1998.

[6] C. I. Henschke, D. I. McCauley, D. F. Yankelevitz, et al., "Early lung cancer action project: overall design and findings from baseline screening," *Lancet*, vol. 354, pp. 99–105, 1999.

[7] K. Oguchi, S. Sone, K. Kiyono, et al., "Optimal tube current for lung cancer screening with low-dose spiral CT," *Acta Radiologica*, vol. 41, no. 4, pp. 352–356, 2000.

[8] S. Itoh, M. Ikeda, S. Arahata, et al., "Lung cancer screening: minimum tube current required for helical CT," *Radiology*, vol. 215, no. 1, pp. 175–183, 2000.

[9] J. Collins, "CT screening for lung cancer: are we ready yet?" *Wisconsin Medical Journal*, vol. 101, no. 2, pp. 31–34, 2002.

[10] J. Hsieh, "Adaptive streak artifact reduction in computed tomography resulting from excessive x-ray photon noise," *Medical Physics*, vol. 25, no. 11, pp. 2139–2147, 1998.

[11] S. Alenius and U. Ruotsalainen, "Bayesian image reconstruction for emission tomography based on median root prior," *European Journal of Nuclear Medicine*, vol. 24, no. 3, pp. 258–265, 1997.

[12] S. Alenius, U. Ruotsalainen, and J. Astola, "Using local median as the location of the prior distribution in iterative emission tomography image reconstruction," *IEEE Transactions on Nuclear Science*, vol. 45, no. 6 pt 2, pp. 3097–3104, 1998.

[13] P. J. Green, "Bayesian reconstructions from emission tomography data using a modified EM algorithm," *IEEE Transactions on Medical Imaging*, vol. 9, no. 1, pp. 84–93, 1990.

[14] B. R. Whiting, "Signal statistics of x-ray computed tomography," in *Medical Imaging 2002: Physics of Medical Imaging*, vol. 4682 of *Proceedings of SPIE*, pp. 53–60, San Diego, Calif, USA, February 2002.

[15] I. A. Elbakri and J. A. Fessler, "Efficient and accurate likelihood for iterative image reconstruction in x-ray computed tomography," in *Medical Imaging 2003: Physics of Medical Imaging*, vol. 5032 of *Proceedings of SPIE*, pp. 1839–1850, San Diego, Calif, USA, February 2003.

[16] I. A. Elbakri, *X-ray CT image reconstruction using statistical methods*, Ph.D. thesis, The University of Michigan, Ann Arbor, Mich, USA, 2003.

[17] J. Hsieh, *Computed Tomography: Principles, Design, Artifacts, and Recent Advances*, SPIE Press, Bellingham, Wash, USA, 2003.

[18] J. A. Fessler, "Statistical image reconstruction methods for transmission tomography," in *Handbook of Medical Imaging*, J. M. Fitzpatrick and M. Sonka, Eds., vol. 2, pp. 1–70, SPIE Press, Bellingham, Wash, USA, 2000.

[19] H. Erdoğan and J. A. Fessler, "Monotonic algorithms for transmission tomography," *IEEE Transactions on Medical Imaging*, vol. 18, pp. 801–814, 1999.

[20] I. Hsiao, A. Rangarajan, and G. Gindi, "A new convex edge-preserving median prior with applications to tomography," *IEEE Transactions on Medical Imaging*, vol. 22, no. 5, pp. 580–585, 2003.

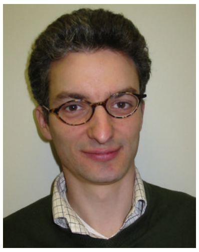

**Patrick J. La Rivière** received the A.B. degree in physics from Harvard University in 1994 and the Ph.D. degree from the Graduate Programs in Medical Physics in the Department of Radiology at The University of Chicago in 2000. He is currently an Assistant Professor in the Department of Radiology at The University of Chicago, where his research interests include tomographic reconstruction in computed tomography, X-ray fluorescence computed tomography, and optoacoustic tomography.

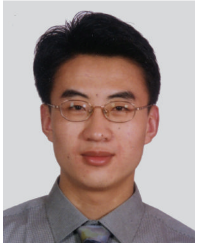

**Junguo Bian** received the B.E. degree from Department of Engineering Physics at Tsinghua University, China, in 1998, and the M.S.N.E. degree from the Department of Nuclear Engineering at Purdue University in 2004. He is currently a Ph.D. student in the Graduate Programs in Medical Physics in the Department of Radiology at The University of Chicago.

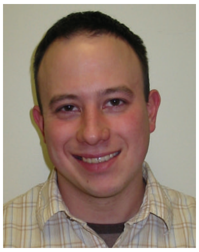

**Phillip A. Vargas** received the B.S. degree in applied physics from Purdue University in 2002. He is currently a Research Technologist in the Department of Radiology at the University of Chicago, where his research interests include tomographic reconstruction in computed tomography, X-ray fluorescence computed tomography, and optoacoustic tomography.

